# Role of Apparent Diffusion Coefficient Value and Apparent Diffusion Coefficient Ratio as Prognostic Factors for Prostate Cancer Aggressiveness

**DOI:** 10.3390/diagnostics14212438

**Published:** 2024-10-31

**Authors:** Arvids Buss, Maija Radzina, Mara Liepa, Edgars Birkenfelds, Laura Saule, Karlis Miculis, Madara Mikelsone, Egils Vjaters

**Affiliations:** 1 Radiology Research Laboratory, Riga Stradins University, LV-1007 Riga, Latvia; mara.liepa@rsu.lv (M.L.); laura.dronka@gmail.com (L.S.); 2 Diagnostic Radiology Institute, Paula Stradina Clinical University Hospital, LV-1002 Riga, Latvia; birkenfelds8@gmail.com; 3 Medical Faculty, University of Latvia, LV-1004 Riga, Latvia; vjaters@gmail.com; 4 Center of Urology, Paula Stradina Clinical University Hospital, LV-1002 Riga, Latvia; karlis.miculis@rsu.lv; 5 Department of Statistics, Riga Stradins University, LV-1007 Riga, Latvia

**Keywords:** ADC value, ADC ratio, prostate cancer, multiparametric MRI, GS, tumor aggressiveness

## Abstract

Background: Prostate cancer is one of the most prevalent cancers in the male population. To determine the aggressiveness of suspected lesions precisely, predictive models are increasingly being developed using quantitative MRI measurements, and particularly the ADC value. This study aimed to determine whether ADC values could be used to establish the aggressiveness of prostate cancer. Methods: A retrospective single-center study included 398 patients with prostate cancer who underwent a multiparametric MRI prior to radical prostatectomy. DWI ADC values were measured (µm^2^/s) using b values of 50 and 1000. The dominant lesion best visualized on MRI was analyzed. The ADC values of the index lesion and reference tissue were compared to tumor aggressivity according to the Gleason grade groups based on radical prostatectomy results. Statistical analysis was performed using the Mann–Whitney U test, Kruskal–Wallis H test, Spearman’s rank correlation, and ROC curves. Results: A very strong negative correlation (*rs* = −0.846, *p* < 0.001) between ADC and GS was found. ROC analysis revealed an AUC of 0.958 and an ADC threshold value of 758 µm^2^/s in clinically significant prostate cancer diagnoses using the absolute ADC value, with no advantage of using the ADC ratio over the absolute ADC value being identified. Conclusion: DWI ADC values and the calculated ADC ratio have a significant inverse correlation with GS. The findings indicate a strong capability in determining prostate cancer aggressiveness, as well as the possibility of assisting with assigning PI-RADS categories using ADC as quantitative metrics.

## 1. Introduction

Prostate cancer (PCa) is one of the most widespread cancers in men worldwide, with prostate cancer alone accounting for 29% of cancer diagnoses in 2023. Prostate cancer can be slow-growing and undiagnosed during a patient’s lifetime, while still attaining excessive numbers of lethal outcomes. In the last few years, significant improvements have been made in early cancer detection due to increased multiparametric magnetic resonance imaging (mpMRI) availability [1].

Today, the aggressiveness of prostate cancer is classified using the International Society of Urological Pathology (ISUP) grade by assigning a Gleason score (GS) to a specific pattern [2]. Th GS is a well-known predictor of prostate cancer aggressiveness. There is no generally agreed upon definition of clinically significant PCa (csPCa); however, on histopathology, it is described as a GS ≥ 7 (including 3+4), volume ≥ 0.5 mL, and/or extra prostatic extension [3].

Traditionally, the diagnostic approach consisted of a blood test of prostate-specific antigen (PSA) and a digital rectal examination, leading to systemic transrectal ultrasound (TRUS)-guided biopsy where only a little and randomly distributed part of the gland is inspected, which results in a considerable risk of both over- and under-sampling. Therefore, a more modern approach includes mpMRI to identify csPCa and reduce possible post-biopsy complications [4]. By prioritizing MRI use, it is possible to avoid unnecessary biopsies and perform them when essential. The PAIREDCAP trial explored this approach, showing that MRI followed by fusion biopsy detected more csPCa (33–38% versus 16%, *p* < 0.001) compared to systemic biopsies [5]. The PRECISION study also found that MRI followed by targeted biopsies detected more significant tumors (38% versus 26%, *p* = 0.005) compared to systemic biopsies [6]. Therefore, MRI has become a cornerstone of PCa diagnosis.

The Prostate Imaging Reporting and Data System (PI-RADS) was introduced in 2012 to improve the identification and likelihood of csPCa. The most important MRI sequence is diffusion weighted imaging (DWI), providing information on tissue composition and tumor cellularity [7]. The b-value determines the DWI sequence sensitivity in identifying the zones of increased diffusivity. Notably, tumor tissue tends to have reduced diffusivity compared to normal tissue due to its increased cellularity; therefore, csPCa on DWI images appear as a high-intensity signal zone.

Therefore, studies have shown that the apparent diffusion coefficient (ADC) derived from DWI seems to correlate inversely with tumor aggressiveness [8,9,10,11,12,13,14]. PI-RADS v2.1 proposes a threshold of 750–900 µm^2^/s to assist in differentiating between benign and malignant prostate tissue, with ADC values below the threshold suggesting a csPCa. However, due to different techniques and variability in ADC values due to non-cancerous lesions and the prostate zone, there are inconsistencies in the acquired ADC measurements. Therefore, no consensus has been reached with respect to an absolute ADC cut-off value [9,13].

A proposed method of overcoming the differences is by using different ADC ratios. The ADC ratio is defined as the ADC tumor value divided by the ADC value of non-cancerous tissue [9]. Several authors have found that the ADC ratio is superior to the absolute ADC value in determination of GS [9,15,16]. However, others have found no benefit to using the ADC ratio [17,18,19]. These various outcomes could be the result of the various reference areas, the multiple MRI systems used, the different reference standards, and the small number of patients.

The aim of this study was to investigate if prostate cancer aggressiveness could be predicted using the ADC value and multiple ADC ratios, using radical prostatectomy specimens as the reference. The hypothesis of our study was the following: higher ADC values are associated with a more aggressive form of PCa and are therefore helpful in the determination of clinically significant and non-significant PCa.

## 2. Materials and Methods

### 2.1. Patient Selection

A retrospective single-center study was conducted including patients from December 2019 till May 2024, who underwent a multiparametric MRI prior to radical prostatectomy (RP). This study was approved by the Research Ethics Commission of Riga Stradins University, Riga, Latvia. The inclusion criteria were as follows: (1) radical prostatectomy conducted in the last 4 years; (2) previously performed multiparametric MRI of prostate with well-defined lesions; and (3) time between RP and mpMRI less than 12 months. Patients were excluded if (1) they received any type of treatment prior to RP; (2) there was poor image quality or lesion not assessable; (3) there were severe artifacts present on the MRI. All the patients received a prostate biopsy prior to RP.

**Figure 1:**
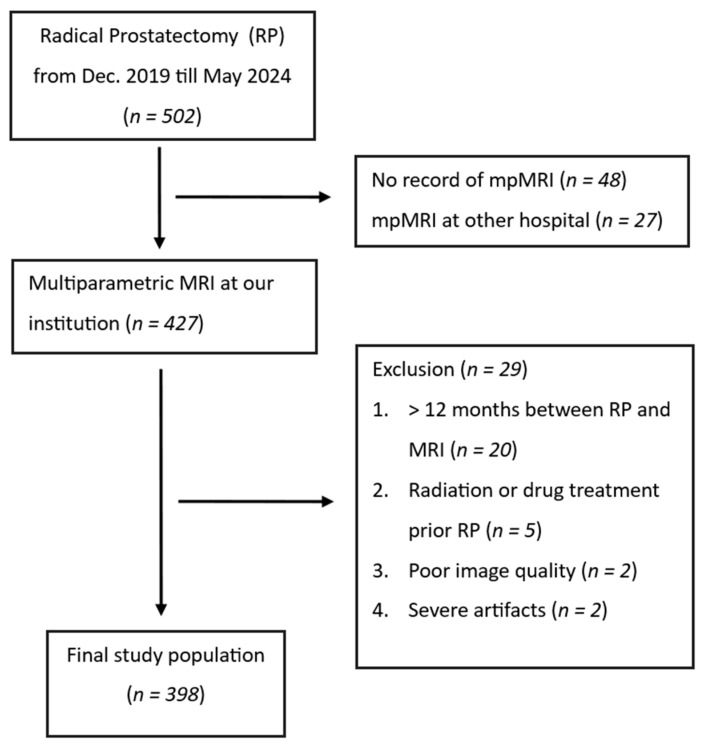
Diagram demonstrating patient selection according to set criteria.

Patient data were acquired from electronic medical records. In the end, 398 patients were chosen for the present study, as presented in Figure 1.

### 2.2. Pathological Examination

The surgical specimens were examined and prepared based on clinical algorithms. All prostate lesions were inspected using hematoxylin and eosin staining by multiple pathologists with different experience levels. Pathological data were acquired from pathology reports, including tumor characteristics. The index lesion was described as the area with the largest volume or extra-prostatic extension. All prostate lesions were described in a structured pathology report, and the location was outlined using a 24-sector map. Prostate total volume was obtained from RP specimens in cc. The pathologists were blinded to any mpMRI measurements.

The GS of every index tumor was separately divided according to ISUP Gleason grade groups. All ISUP grade groups were subdivided into two groups: GS 6, or non-significant prostate cancer (nsPCa); and GS ≥ 7, or clinically significant prostate cancer (csPca). However, for sub-analysis, we also included GS 7(3+4) in the nsPCa group, creating two different groups: GS 6, 7(3+4) and GS ≥ 7(4+3).

### 2.3. MRI Technique

Multiparametric MRI of the prostate performed on a MAGNETOM Sola Siemens 1.5T system in Pauls Stradins Clinical University hospital in Diagnostic Radiology Institute. MRI protocol included axial T1-weighted turbo spin-echo images; axial, sagittal, and coronal T2-weighted turbo spin-echo images; diffusion weighted images using three b-value gradients of 150, 800, and 1200 s/mm^2^; a calculated ADC map; and a transverse dynamic contrast series. Imaging protocol details listed in Table 1.

### 2.4. Image Analysis and Data Collection

All acquired images were analyzed by two radiologists with 10 years of experience in reading prostate MRI. The picture archiving and communication system Sectra IDS7 was used to review all examinations. As only the dominant lesion with the best visualization was inspected, the two readers matched the index lesion on the MRI with the lesion in the surgical specimen using histopathology reports and a 24-sector map, albeit blinded to GS and other characteristics.

In the following image examination and interpretation, each reader documented the prostate volume, lesion maximum diameter, prostate zone (PZ, TZ), and PI-RADS score. To obtain ADC, two b-values were used: 50 and 1000 s/mm^2^. Both readers measured each lesion’s ADC using region of interest (ROI) on the slice with the largest cross-sectional area of the lesion. The ROI was not fixed and was drawn as a circle to include most of the index lesion, without any surrounding prostate tissue. Additionally, three separate ROIs of at least 20 mm^2^ were placed in peripheral zone (PZ), transitional zone (TZ), and urinary bladder (UB) for reference. For each ROI, the mean ADC value was noted. Three ADC ratios were calculated based on location of reference: (1) tumor to PZ; (2) tumor to TZ; and (3) tumor to UB.

**Figure 2:**
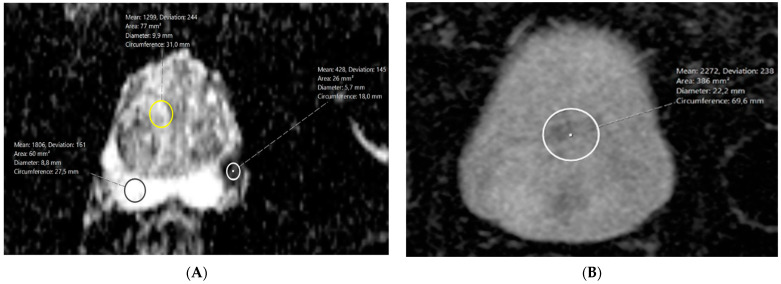
(**A**) Circular ROIs drawn in tumor (white), non-cancerous transitional zone (yellow), and noncancerous peripheral zone (black). Tumor ADC = 428 µm^2^/s; TZ ADC = 1299; and PZ ADC = 1806. (**B**) Circular ROI drawn in homogenous area of urinary bladder on ADC map. Urinary bladder ADC = 2272 µm^2^/s.

Figure 2 shows a representative case of a ROI placement in a 74-year-old patient, PSA 11.9 ng/mL, classified as PI-RADS 5 on MRI and GS 8 on histopathology from a surgical specimen.

### 2.5. Statistical Analysis

Statistical analysis was performed using SPSS (IBM SPSS Statistics for Windows, Version 29.0: IBM Corp). Descriptive statistics were used for demographic data. Quantitative data analysis between two groups was conducted using the Mann–Whitney U test, and for multiple groups, the Kruskal–Wallis H test was used. Spearman’s rank correlation (r) was used to evaluate the association between the absolute ADC, ADC ratio, and GS.

Receiver operating characteristic (ROC) curves were used, and area under the curve (AUC) was calculated to evaluate the ADC and ADC ratio’s ability to distinguish between GS 6 and GS ≥ 7 and GS ≤ 7(3+4) and GS ≥ 7(4+3) and to acquire the ADC threshold value.

## 3. Results

The patient characteristics are displayed in Table 2. The tumor characteristics included PI-RADS, GS, pathological T-stage, and lesion location. The results for lesions per cohort with relative percentage were as follows: PI-RADS 2 (21/398; 5%); PI-RADS 3 (39/398; 9%); PI-RADS 4 (158/398; 40%); and PI-RADS 5 (181/398; 46%); ISUP 1 (97/398; 24%); ISUP 2 (188/398; 48%); ISUP 3 (70/398; 17%); ISUP 4 (24/398; 6%); and ISUP 5 (19/398; 5%); and pathological T-stage pT2a (35/398; 8%), pT2b (10/398; 3%), pT2c (213/398; 54%), pT3a (99/398; 25%), and pT3b (41/398; 10%).

Most index lesions classified as GS 3+4 (363/398; 91%) were located in the PZ and only (35/398; 9%) located in the TZ.

The ADC tumor and ADC ratio results compared between nsPCa and csPCa are summarized in Table 3. The results showed a statistically significant difference (*p* < 0.001) between the non-cancerous region ADC and the ADC value of the index lesion. However, there was no statistically significant difference (*p* > 0.05) between non-cancerous tissue in nsPCa and csPCa, showing equivalent results both in PZ and TZ, as well as in UB.

Regarding the ADC tumor and the ADC ratio, results appeared significantly lower in patients with csPCa (*p* < 0.001). In contrast to non-cancerous ADC values, the ADC ratio displayed the highest results in the TZ but the lowest in the UB.

Review of data displayed a characteristic distribution of ADC values depending on GS—ADC median ≥ 880 µm^2^/s and ADC mean ≥ 874 µm^2^/s for GS 6; ADC median 689 µm^2^/s and ADC mean ranging from 693 to 710 µm^2^/s for GS 7(3+4); ADC median 634 µm^2^/s and ADC mean ranging from 622 to 650 µm^2^/s for GS 7(4+3); ADC median 578 µm^2^/s and ADC mean ranging from 558 to 594 µm^2^/s for GS 8; and ADC median ≤ 540 µm^2^/s and ADC mean ≤ 532 µm^2^/s for GS 9,10. However, GS 6 and both 7’s appeared to have overlapping values in 28/398 (7%) cases.

Spearman’s rank correlation displayed a very strong negative correlation between ADC tumor and GS, with the highest result being rs = −0.832 with a significance level of *p* < 0.001. However, the ADC ratio of tumor to PZ, tumor to TZ, and tumor to UB showed inferior results, with rs = −0.537, −0.531, and −0.667, respectively.

**Figure 3:**
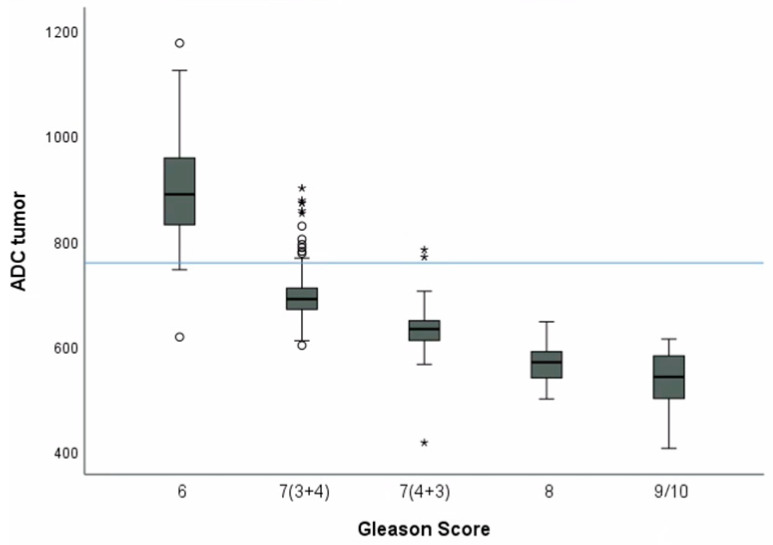
Box and whisker plots of ADC tumor correlation with GS based on RP. ADC threshold of 758 µm^2^/s (blue line). Outliers marked with stars and circles.

Using the Mann–Whitney U test, we found statistically significant differences (*p* < 0.001) only between GS 6 and 7(3+4), GS 7(3+4), and 7(4+3). Although there was no statistically significant difference (*p* > 0.05) between GS 7(4+3) and 8 and 9–10, ADC values showed a gradual decrease between these groups (Figure 3).

**Figure 4:**
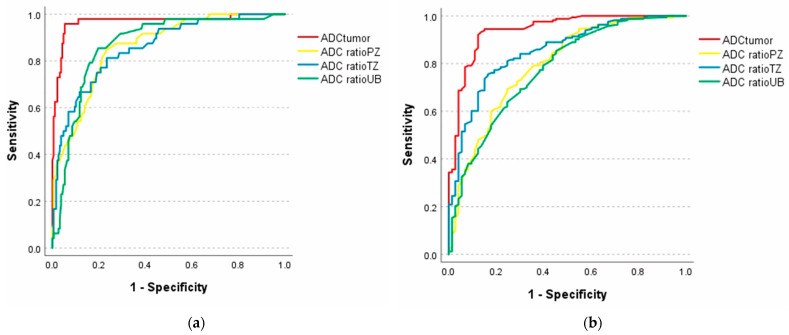
(**a**) ROC curve analysis of ADC measurements between GS 6 and GS ≥ 7. (**b**) ROC curve analysis of ADC measurements between GS ≤ 7(3+4) and GS ≥ 7(4+3).

The ROC curve analysis proposed an ADC threshold value of 758 µm^2^/s to differentiate between non-significant and csPCa, showing the highest AUC of 0.958, with sensitivity and specificity of 90.7% and 89.4%, respectively. However, including GS 7(3+4) in the non-significant group displayed similar results, with an ADC threshold of 651 µm^2^/s, AUC of 0.926, sensitivity of 90.9%, and specificity of 84.1%, as demonstrated in Figure 4.

The three different ADC ratio values showed inferior results to the ADC lesion in predicting csPCa, with AUC of 0.863, 0.853, and 0.879. Furthermore, comparing the ADC ratio with GS 7(3+4) in both the nsPCa and csPCa groups displayed marginally lower AUC results. However, the ADC tumor-to-TZ ratio displayed equivalent results with GS 7(3+4), being in both non-significant and significant groups, with AUC of 0.852 and 0.853, respectively. Detailed results are displayed in Table 4.

## 4. Discussion

Our presented retrospective single-center study shows an inverse correlation between the absolute ADC, ADC ratio, and GS of prostate cancer based on radical prostatectomy results. This outcome concurs with previous studies that found an inverse correlation between ADC and GS.

The definition of csPCa is essential when comparing the results of various studies as most authors attempt to define the ADC threshold value in relation to tumor aggressiveness. Some authors define GS 6 as non-significant and GS ≥ 7 as clinically significant [7,8,17,18,19,20], whereas others include GS 7(3+4) in the non-significant group. Boesen et al. conducted a study including GS 7(3+4) in both non-significant and significant groups to obtain two different cut-offs. Their results displayed an increase in AUC from 0.80 with GS 7(3+4) in the clinically significant group to 0.90 for the ADC_ratio_ with GS 7(3+4) in the non-significant group [12]. However, this contrasts with our results, as we found no improvement between both groups, as well as showing noticeably lower AUC for the ADC_ratio_. Moreover, in comparing ADC values between all Gleason groups, the GS 7(3+4) tends to have much lower ADC values than GS 6 (ADC median 689 vs. 880 µm^2^/s, respectively), suggesting its significance as an intermediate risk of prostate cancer, and it should therefore be classified as clinically significant.

Additionally, studies comparing the ADC and the ADC ratio observed that the ADC ratio—mostly tumor versus non-cancerous contralateral tissue—was preferable in differentiating significant from non-significant PCa, with reported AUCs levels of 0.80–0.85 versus 0.71–0.73 [13,15,21,22]. However, other authors found no advantage in ADC ratio compared to ADC [17,18,19], though some only discovered similar a performance between the ADC and the tumor-to-UB ADC ratio, with AUC 0.96 vs. 0.93 and 0.794 vs. 0.790, respectively [18,23]. In our study, we found no added value or advantage in using the ADC ratio; in fact, our results indicate that the absolute ADC value offers greater results in differentiating low-risk and high-risk tumors (AUC 0.958 vs. 0.777), regardless which of the three different reference areas is used. Even so, no noticeable difference was observed between the three reference areas (AUC 0.777 vs. 0.743 vs. 0.755), although other authors seem to have found the tumor-to-UB ADC ratio to be superior to the other ADC ratios. Itatani et al. [16] even used the internal obturator muscle as the ADC reference; however, the AUC of the muscle was equivalent to that of the absolute ADC. We excluded nearby muscle as a reference as few studies suggest that ADC values in muscle could vary due to some factors, like patients age, muscle development, and some muscle disorders [24,25,26].

The concept of the ADC ratio seems a more useful method as it could yield helpful results with respect to different MRI systems and *b*-values. The use of ADC alone raises a concern, considering how different *b*-values could affect absolute ADC values [9]. However, Woo et al. [18] pointed out various reasons why using the ADC value for internal references could not yield helpful ADC ratios. One concern they also noted is the post-biopsy hemorrhage, which could modify the signal intensity on the T2-weighted image, DWI, and ADC map, lasting up to 8 weeks. Furthermore, they highlight that non-cancerous tissue in PZ can vary according to age, and the intrinsically heterogeneous nature of TZ can exhibit different ADC values. Surov et al. [27] also pointed out that the presence of benign prostate hyperplasia, exclusively within TZ, showed lower ADC values. Moreover, it is thought that urine can show different characteristics depending on patient-related factors (i.e., urine composition, urine specific gravity), therefore causing variations in ADC measurements. We also noticed that the amount of urine influenced the ADC value, meaning that a low amount or no urine showed ADCs below ~1600 µm^2^/s; however, those with a partially or fully filled urinary bladder would have ADC values reaching as high as 4000 µm^2^/s. DeCobelli et al. [17] hypothesized that possible prostate tissue changes such as chronic inflammation and fibrosis in patients with more aggressive PCa also affect the ADC measurements. Boesen et al. also proposed some concerns about the ROI placement as different-sized ROI could alter the acquired ADC value as it may not address the full heterogeneity of the tumor [13]. However, one study found no statistically significant difference in ADC values between three the different ROIs (free hand, large-circle, and small-circles) [28].

Our study has several limitations. First, the retrospective design of our study could have caused a selection bias. Furthermore, as our goal was also to evaluate different ADC ratios in determining GS, the retrospective aspect restricted us from using another reference, i.e., an external saline bottle. In a study conducted by Itatani et al. [16], one of the references for the ADC ratio was the externally placed saline bottle, which showed greater results in determining csPCa (AUC = 0.846 vs. 0.711) compared to non-cancerous prostate regions and the urinary bladder. However, as the ADC_tumor_ values in our study displayed superior results over ADC_ratio_, we think that using an external saline bottle, which would mostly show a constant ADC value, may not, in fact, yield any improvement and would be equivalent to that of the absolute ADC [18].

Second, histopathology reports were reported by different pathologists with different experience levels. Also, most of the reports did not include information about the percentage of the Gleason pattern, which is especially important for GS 3+4, meaning that a lower percentage of Gleason 4 could have caused a higher ADC value, thereby assigning them the status of non-significant PCa.

Third, the fact that we only focused on the best-visualized and most-aggressive lesion (index lesion) of prostate cancer, ensuring that ROIs would not include non-cancerous tissue, our study may have offered the best-case scenario, with less-accurate measurements being likely in actual clinical practice. Additionally, multiparametric MRI is not able to detect all possible cancer foci [29]; furthermore, the accuracy in detecting and locating prostate cancer somewhat depends on the affected region [30].

## 5. Conclusions

In conclusion, a clear inverse correlation between ADC values, the ADC ratio, and prostate cancer aggressiveness is evident; however, the absolute ADC value proves to be more valuable than the ADC ratio. This negative relation implies that a decrease in ADC values is associated with an increased probability of malignancy in prostate lesions. ADC values display a remarkable sensitivity and specificity in the assessment of cancer aggressiveness. The diagnostic potential of ADC values is notably high and may serve as a quantitative metric to assist in the assigning of PI-RADS categories. Additionally, it serves as an approach to preventing unnecessary biopsies and treatments, thereby assisting clinicians in devising more refined treatment strategies and informed prognosis assessments. Therefore, radiologists are advised to prioritize the integration of ADC measurements to increase diagnostic precision. However, it is important to recognize the limitations when evaluating study outcomes; thus, prospective multicenter studies with a standardized MRI protocol are mandatory.

## Figures and Tables

**Table 1:** MRI scan protocol.

Parameters	Sequence	TR (ms)	TE (ms)	Slice (mm)	FOV (cm)
Axial T2W	TSE	7690	100	3	20
Sagittal T2W	TSE	7000	100	3	20
Coronal T2W	TSE	7250	100	3	20
Axial T1W	TSE	700	10	4	20
Axial DCE	VIBE	4.2	2.3	3	24
Axial DWI b = 50,800,1200 s/mm^2^	EPI	5600	74	3	20

**Table 2:** Patient characteristics.

Parameter	Mean ± SD (Min–Max)
Age, years	65.4 ± 6.5 (44–79)
Time between MRI and RP, months	3.9 ± 2.3 (1–11)
PSA, ng/mL	10.77 ± 9.83 (0.83–98.9)
PSA density, ng/mL^2^	0.23 ± 0.01 (0.03–1.75)
RP prostate volume, cc	50.87 ± 20.03 (17.57–131.80)
MRI lesion, mm	14.75 ± 6.81 (3–40)

**Table 3:** Comparison of ADC_tumor_ and ADC_ratio_ for prostate cancer.

Variable	GS 6	GS ≥ 7(3+4)	*p*
**ADC (** **µm^2^/s)**			
Peripheral zone			
(mean ± SD; range)	1649 ± 293 (1020–2450)	1602 ± 289 (718–2320)	0.624
(median; IQR)	1632 (1450–1840)	1605 (1387–1810)	
Transitional zone			
(mean ± SD; range)	1281 ± 228 (942–1879)	1290 ± 227 (716–2040)	0.472
(median; IQR)	1252 (1106–1401)	1275 (1140–1430)	
Urinary bladder			
(mean ± SD; range)	2526 ± 422 (1613–3992)	2439 ± 471 (1044–3721)	0.582
(median; IQR)	2559 (2188–2800)	2512 (2135–2761)	
Index lesion			
(mean ± SD; range)	875 ± 93 (617–1176)	666 ± 78 (405–965)	<0.001
(median; IQR)	880 (812–906)	669 (624–700)	
**ADC ratio**			
Tumor to peripheral zone			
(mean ± SD; range)	0.56 ± 0.11 (0.36–0.91)	0.42 ± 0.09 (0.24–0.84)	<0.001
(median; IQR)	0.54 (0.49–0.64)	0.42 (0.36–0.49)	
Tumor to transitional zone			
(mean ± SD; range)	0.72 ± 0.13 (0.43–1.03)	0.52 ± 0.11 (0.27–0.96)	<0.001
(median; IQR)	0.74 (0.68–0.9)	0.51 (0.45–0.60)	
Tumor to urinary bladder			
(mean ± SD; range)	0.37 ± 0.07 (0.20–0.56)	0.28 ± 0.07 (0.15–0.63)	<0.001
(median; IQR)	0.35 (0.31–0.40)	0.27 (0.24–0.31)	

**Table 4:** ROC curve analyses comparing ADC_tumor_ and ADC_ratio_ to determine csPCa.

Variable	Threshold Value	AUC	Sensitivity	Specificity	*p*
**GS 6 vs. ≥ 7**					
ADC tumor (µm^2/^s)	≤758	0.958	90.7	89.4	<0.001
ADC ratio PZ	≤0.48	0.863	83.3	79.7	<0.001
ADC ratio TZ	≤0.60	0.853	81.3	76.6	<0.001
ADC ratio UB	≤0.30	0.879	85.4	80.3	<0.001
**GS 6 and 7(3+4) vs. ≥7(4+3)**					
ADC tumor (µm^2^/s)	≤651	0.926	90.9	84.1	<0.001
ADC ratio PZ	≤0.38	0.793	80.4	62.6	<0.001
ADC ratio TZ	≤0.50	0.852	81.0	73.3	<0.001
ADC ratio UB	≤0.25	0.793	79.1	60.3	<0.001

## Data Availability

Data are available from the corresponding author after an appropriate review.
